# Annual Meeting of the Massachusetts Medical Society

**Published:** 1871-06

**Authors:** 


### Annual Meeting of the Massachusetts Medical Society.

We chanced to be in Boston the first day of the meeting of this Society, and took much pleasure in looking in upon its proceedings. We found the New York State Medical Society most ably represented by Drs. Hutchinson of Brooklyn, and Kneeland of Onondaga, and were happy that we had nothing to do except as a private citizen, to observe the workings of this ancient and honorable Society. Papers were read by the followitg physicians:—

Dr. Edward Wigglesworth of Boston, on *Baldness.*

Dr. Henry Tuck, Boston, *Torsion of Bloodvessels.*

Dr. R. H. Fitz, Boston, *Tuberculosis.*

Dr. Wm. L. Richardson, Boston, *External Manipulation in Obst trics.*

Dr. H. J. Bowditch, Boston, *Venisection.*

We were not present during the reading of any of these papers except the one on Torsion of Bloodvessels, and this was a well written paper upon the subject, showing that torsion, if properly made, can be trusted for arresting hemorrhage in vessels of the largest class.

A visit to the city Hospital offered opportunity to observe several minor operations in surgery, which were made by the attending surgeons. Ether was used as the Anæsthetic; was administered prior to the patient's being brought to the operating room, and was continued in some cases, no apparent notice being taken of the livid appearance and stertorious breathing of patients. So far as the administration of ether is concerned, and its apparent effect upon the patients, the exhibition was far from attractive.

The Editor of the Boston *Medical and Surgical Journal*, not long since, thought that a surgeon who should be guilty of administering chloroform, was justly chargeable with manslaughter. If it was given with the apparent recklessness that ether is given in Boston, we quite agree with him. We returned home with the conviction, that chloroform, with careful watching, was safer, more pleasant, and every way to be preferred to sulphuric ether as administered in the city claiming to have made its discovery.

Of the second days proceedings we have no personal knowledge, but learn from reliable sources that the grand features of the meeting were comprised in the scientific papers, Annual address of Dr. Bigelow, and Poem by Dr. T. Stone, of Wellfleet.

